# *SPTLC2* variants are associated with early‐onset ALS and FTD due to aberrant sphingolipid synthesis

**DOI:** 10.1002/acn3.52013

**Published:** 2024-02-05

**Authors:** Hiroya Naruse, Hiroyuki Ishiura, Kayoko Esaki, Jun Mitsui, Wataru Satake, Peter Greimel, Nanoka Shingai, Yuka Machino, Yasumasa Kokubo, Hirotoshi Hamaguchi, Tetsuya Oda, Tomoko Ikkaku, Ichiro Yokota, Yuji Takahashi, Yuta Suzuki, Takashi Matsukawa, Jun Goto, Kishin Koh, Yoshihisa Takiyama, Shinichi Morishita, Takeo Yoshikawa, Shoji Tsuji, Tatsushi Toda

**Affiliations:** ^1^ Department of Neurology, Graduate School of Medicine The University of Tokyo Tokyo Japan; ^2^ Department of Precision Medicine Neurology, Graduate School of Medicine The University of Tokyo Tokyo Japan; ^3^ Department of Neurology Okayama University Graduate School of Medicine, Dentistry and Pharmaceutical Sciences Okayama Japan; ^4^ Department of Biotechnology and Life Sciences, Faculty of Biotechnology and Life Sciences Sojo University Kumamoto Japan; ^5^ Laboratory for Cell Function Dynamics, RIKEN Centre for Brain Sciences Wako Saitama Japan; ^6^ Division of Applied Life Science, Graduate School of Engineering Sojo University Kumamoto Japan; ^7^ Department of Neurology National Hospital Organization Mie National Hospital Tsu Mie Japan; ^8^ Kii ALS/PDC Research Center, Graduate School of Regional Innovation Studies Mie University Tsu Mie Japan; ^9^ Department of Neurology Kita‐Harima Medical Center Ono Hyogo Japan; ^10^ Division of Neurology Kobe University Graduate School of Medicine Kobe Hyogo Japan; ^11^ Department of Neurology Hyogo Prefectural Rehabilitation Central Hospital Kobe Hyogo Japan; ^12^ Department of Neurology National Hospital Organization Hyogo‐Chuo National Hospital Sanda Hyogo Japan; ^13^ Department of Neurology National Center Hospital, National Center of Neurology and Psychiatry Tokyo Japan; ^14^ Department of Computational Biology and Medical Sciences, Graduate School of Frontier Sciences The University of Tokyo Chiba Japan; ^15^ Department of Neurology International University of Health and Welfare Ichikawa Hospital Chiba Japan; ^16^ Department of Neurology, Graduate School of Medical Sciences University of Yamanashi Yamanashi Japan; ^17^ Department of Neurology Yumura Onsen Hospital Yamanashi Japan; ^18^ Department of Neurology Fuefuki Central Hospital Yamanashi Japan; ^19^ Laboratory of Molecular Psychiatry, RIKEN Center for Brain Science Wako Saitama Japan; ^20^ Institute of Medical Genomics International University of Health and Welfare Chiba Japan

## Abstract

**Objective:**

Amyotrophic lateral sclerosis (ALS) is a devastating, incurable neurodegenerative disease. A subset of ALS patients manifests with early‐onset and complex clinical phenotypes. We aimed to elucidate the genetic basis of these cases to enhance our understanding of disease etiology and facilitate the development of targeted therapies.

**Methods:**

Our research commenced with an in‐depth genetic and biochemical investigation of two specific families, each with a member diagnosed with early‐onset ALS (onset age of <40 years). This involved whole‐exome sequencing, trio analysis, protein structure analysis, and sphingolipid measurements. Subsequently, we expanded our analysis to 62 probands with early‐onset ALS and further included 440 patients with adult‐onset ALS and 1163 healthy controls to assess the prevalence of identified genetic variants.

**Results:**

We identified heterozygous variants in the serine palmitoyltransferase long chain base subunit 2 (*SPTLC2*) gene in patients with early‐onset ALS. These variants, located in a region closely adjacent to ORMDL3, bear similarities to *SPTLC1* variants previously implicated in early‐onset ALS. Patients with ALS carrying these *SPTLC2* variants displayed elevated plasma ceramide levels, indicative of increased serine palmitoyltransferase (SPT) activity leading to sphingolipid overproduction.

**Interpretation:**

Our study revealed novel *SPTLC2* variants in patients with early‐onset ALS exhibiting frontotemporal dementia. The combination of genetic evidence and the observed elevation in plasma ceramide levels establishes a crucial link between dysregulated sphingolipid metabolism and ALS pathogenesis. These findings expand our understanding of ALS's genetic diversity and highlight the distinct roles of gene defects within SPT subunits in its development.

## Introduction

Amyotrophic lateral sclerosis (ALS) is an intractable neurodegenerative disorder characterized by selective motor neuron degeneration, which leads to progressive motor function loss and typically results in death within 3–5 years. While most ALS cases are sporadic (SALS), approximately 5%–10% are familial (FALS).^1^, ^2^ Although over 40 genes have been implicated in ALS,^3^, ^4^ a comprehensive understanding of the underlying pathogenesis remains elusive, with no curative treatments currently available.

Our research, focusing on the monogenic basis of ALS, identified pathogenic variants in ALS‐causing genes in 59% of FALS and 3.6% of SALS in a Japanese series.^5^, ^6^, ^7^ Despite the discovery of numerous ALS‐causing genes, a definitive genetic cause remains elusive for a substantial proportion of cases, especially in SALS. This lack of clarity on genetic causes underscores the urgent need for identifying additional ALS‐causing genes to improve our understanding of ALS pathogenesis and to facilitate the development of rational therapeutic strategies.

Patients with ALS typically present with initial symptoms at 55–75 years of age, with most cases occurring after the age of 40 years.^8^ However, there have been reports of ALS manifesting in individuals aged 25 years or younger (juvenile ALS) and in those aged 40 years or younger (early‐onset ALS).^9^ Such early‐onset ALS cases often display a variable clinical course and are sometimes distinct from typical ALS. Despite the relatively low incidence of early‐onset ALS, its genetic predisposition is estimated to be greater than that of typical ALS, making these cases a crucial target for genetic analysis to discover new ALS‐causing genes.^9^

In this study, we analyzed whole‐exome sequencing data from Japanese patients with early‐onset ALS to identify previously unreported genetic etiologies of the disease. We discovered two previously unknown variants in the serine palmitoyltransferase long chain base subunit 2 (*SPTLC2*; NM_004863) gene, specifically located within its membrane‐associated region: one in a family with early‐onset FALS and another as a de novo variant in a patient with SALS. We described the clinical and genetic features of four patients from two independent families carrying dominantly acting, monoallelic, *SPTLC2* variants. Plasma lipid analysis revealed elevated ceramide levels in patients with *SPTLC2*‐ALS compared to healthy individuals, indicating increased serine palmitoyltransferase (SPT) activity. Our study establishes *SPTLC2* variants linked to early‐onset ALS with frontotemporal dementia (FTD), underscoring their pivotal role in the disease's pathology. Recent reports have linked *SPTLC1* variants with early‐onset ALS.^10^, ^11^ Among these reports, some specifically highlighted elevated ceramide levels as a contributing factor,^10^ a notion supported by observations of increased ceramide levels in the plasma and spinal cords of patients with SALS.^12^ These findings emphasize the integral role of lipid metabolism disturbances in ALS pathogenesis.

## Methods

### Participants with early‐onset ALS

Patients diagnosed with early‐onset ALS and their family members were recruited for our clinical and molecular genetic studies. The study was conducted according to the Declaration of Helsinki, with approval from the Institutional Review Board of the University of Tokyo (protocol code, G1396). All the participants provided written informed consent. Patients were diagnosed with clinically definite, probable, laboratory‐supported probable, or possible ALS based on the revised El Escorial criteria.^13^

Sixty‐two early‐onset ALS probands from our facility were enrolled: 24 probands with FALS and 38 patients with SALS, each with an onset age of <40 years. Prior to this study, all patients had been screened for variants or expansions of known ALS‐associated genes. For the genes *SOD1, FUS, TARDBP, SETX, VAPB, SPG11, OPTN, VCP, UBQLN2, ALS2, SIGMAR1, PFN1, ERBB4, HNRNPA1, HNRNPA2B1, MATR3, ANXA11, TBK1, KIF5A, TIA1, GLT8D1, SPTLC1, CCNF, CYLD, ANG, FIG4, CHMP2B, TUBA4A, NEK1, DAO, SPAST, GLE1, SS18L1, CHCHD10, SQSTM1, GRN, MAPT, DCTN1, ERLIN2, ERLIN1, HSPB1, ATP13A2, DJ1, CACNA1H, SORD, SYNE1, VRK1, PNPLA6, NEFH, EWSR1, PRPH*, and *TAF15*,^14^ we utilized whole‐exome sequence data to identify rare variants. These variants included missense, nonsense, splice‐site, and short insertion/deletion variants with a minor allele frequency (MAF) <1%, as referenced from the public population databases gnomAD and ToMMo (jMorp, 38KJPN). Repeat expansion mutations in *C9ORF72* were detected using repeat‐primed PCR. Intermediate CAG repeat expansions in *ATXN2* were confirmed by fragment analysis.

**Figure 1:**
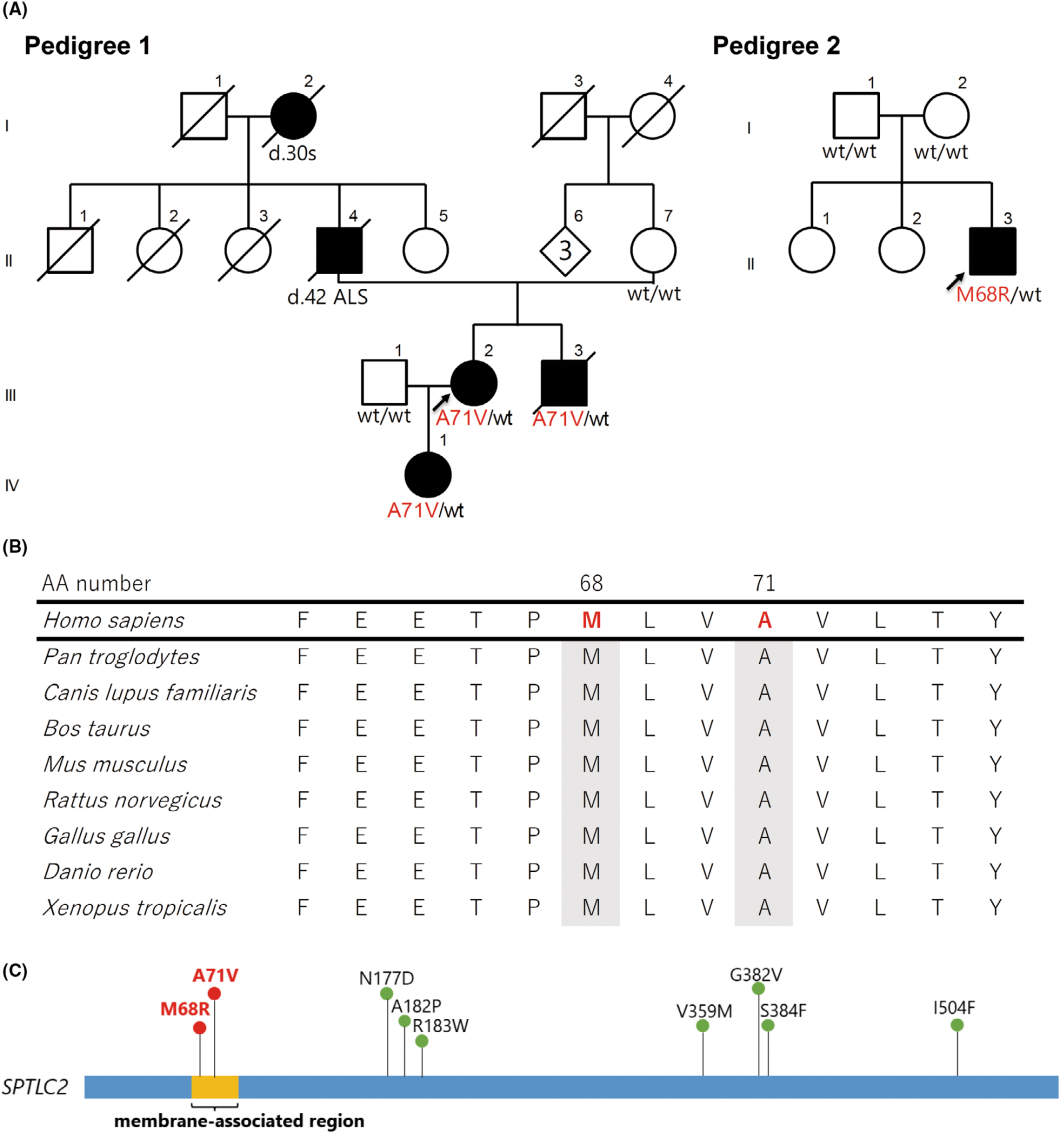
Identification of *SPTLC2* variants in patients with early‐onset ALS. (A) Pedigree charts of patients with early‐onset ALS carrying *SPTLC2* variants. The variant alleles in *SPTLC2* are shown in red, along with their specific variant, while wild‐type alleles are denoted by “wt.” Arrows indicate probands. Filled and open symbols represent ALS‐affected and ALS‐unaffected individuals, respectively, and slashed symbols indicate deceased individuals. Squares represent males, circles denote females, and rhomboids indicate the number of family members of an unknown sex. (B) Evolutionary conservation of altered SPTLC2 amino acids associated with early‐onset ALS. “AA number” refers to the amino acid position within the human SPTLC2 protein. (C) Schematic representation of the SPTLC2 protein. Variants associated with early‐onset ALS (red) identified in the present study were located within the membrane‐associated region of SPTLC2, in contrast to those associated with HSAN1 (green).

Through our mutational analyses, pathogenic variants in ALS‐associated genes were identified in 20 probands with FALS: 13 had a variant in *SOD1*, 5 in *FUS*, 1 in *VCP*, and 1 in *HNRNPA1*. Among the patients with SALS, pathogenic variants were found in five: 3 had a variant in *SOD1*, 1 in *SETX*, and 1 in *SYNE1*.^15^ Consequently, 37 probands with early‐onset ALS—comprising four probands with FALS and 33 patients with SALS—without identified known pathogenic variants proceeded to further investigation. Additionally, we included 3 affected and 2 unaffected individuals from Pedigree 1, as well as the proband and his unaffected parents from Pedigree 2 (Fig. 1A).

### Whole‐exome sequencing and variant analysis

Genomic DNA was extracted from peripheral blood leukocytes obtained from the participants using standard procedures. Whole‐exome sequencing was performed according to a previously established workflow.^6^ Briefly, the SureSelect Human All Exon v6 + UTRs kit (Agilent, Santa Clara, CA) was used to enrich exon sequences, which were then sequenced using an Illumina Hiseq2500 platform (Illumina, San Diego, CA) according to the manufacturer's instructions. Sequencing data alignment (GRCh37/hg19) and variant calling were conducted using the Burrows–Wheeler Aligner and SAMtools. Variants were confirmed by direct nucleotide sequencing using an ABI 3730 Genetic Analyzer.

For Pedigree 1, whole‐exome sequencing data were analyzed to identify rare variants (missense, nonsense, splice‐site, and insertion/deletion) present in affected individuals and absent in unaffected individuals. During the variant filtering process, public population databases, including dbSNP, gnomAD, and ToMMo (jMorp, 38KJPN), were referenced (last accessed October 2022), along with an in‐house database of 1163 healthy Japanese control exomes (Fig. S1).

### Identity by descent analysis

In confirming the biological relationship between the proband and their parents in Pedigree 2, we assessed the pairwise identity by descent (IBD) between the proband and each parent by analyzing the whole‐exome data using PLINK 1.90.^16^ For IBD calculation, we selected 25,612 exonic SNPs after excluding SNPs with a minor allele frequency (MAF) <0.1 or >0.9 (in ExAC v1) or an average read depth < 20×. The PI_HAT values calculated for the proband‐father and proband‐mother pairs were 0.5181 and 0.5348, respectively, confirming the paternity and maternity of the proband's parents.

### Trio analysis

Trio analysis was conducted specifically in pedigree 2. We examined de novo variants that were present in the proband but absent in the parents based on whole‐exome data through the following procedure. First, the gVCF files for the trio were merged and jointly genotyped using GATK 4.4. Next, candidate de novo variants were searched using Triodenovo (v0.06) with the parameters “‐‐minDQ 10 ‐‐minDepth 10,” which means the de novo mutation model must be favored over the Mendelian inheritance model by a log‐likelihood ratio >10.^17^

Subsequently, we applied the basic filtering strategy recommended by the program authors. The retained variants were biallelic, had a QUAL ≥30, and exhibited homozygous reference genotypes in the parents and heterozygous genotypes in the child, with the heterozygous phred‐scaled genotype likelihood (PL) being zero and the minimum PL of the other two genotypes in offering to be 30 (i.e., a heterozygous call is favored by a log‐likelihood ratio >30).

### Protein structure analysis

A cryo‐electron microscopic structure of the dimeric SPT/ORM1‐like protein 3 (ORMDL3) complex was generated and examined utilizing the Protein Data Bank Japan (PDBj) database to understand better the identified *SPTLC2* variants' structural implications. The complex structure was visualized using the Molmil web‐based molecular viewer.^18^ Our structural analysis was conducted based on the PDB entry 6M4N.^19^

### Sphingolipid measurements

Lipid analyses were specifically performed on plasma samples from two pedigrees with early‐onset ALS identified in this study. For Pedigree 1, samples from two affected individuals and one unaffected family member were analyzed. In Pedigree 2, the analysis included samples from one affected individual and both unaffected parents. Sphingolipids were directly extracted from the samples and quantified utilizing liquid chromatography‐electrospray ionization‐tandem mass spectrometry (LC‐ESI‐MS/MS) as outlined in Supplementary Methods.^20^ Each condition and variant underwent three to four independent measurements.

### Participants and mutational analysis for adult‐onset ALS cases and controls

We analyzed 40 probands with FALS and 400 patients with SALS with adult‐onset ALS (age at onset ≥40 years). These patients did not possess pathogenic variants of known ALS‐associated genes, as confirmed by our mutational analyses, which included whole‐exome sequencing and repeat‐primed PCR analysis. Concurrently, we used whole‐exome sequencing data from 1163 unrelated healthy Japanese individuals without a history of ALS or FTD as controls. The frequency of rare *SPTLC2* variants was investigated by whole‐exome sequencing.

### Statistics

All statistical evaluations of sphingolipid analysis were conducted using GraphPad Prism version 9 (GraphPad Software, Inc.). The Shapiro–Wilk test confirmed the normality of the data. Given the presence of heteroscedasticity (*p* < 0.05), as determined by the Brown–Forsythe test, analysis of variance (ANOVA) with Welch's adjustment was performed across all conditions. When ANOVA identified significant differences, we employed Dunnett's multiple comparison tests for subsequent post hoc analysis. An adjusted *p*‐value of <0.05 was considered statistically significant.

## Results

### Identification of a *SPTLC2* variant in a family with early‐onset FALS

We initially investigated a four‐generation family with early‐onset ALS, displaying an autosomal dominant inheritance pattern (pedigree 1; Fig. 1A). Given the early disease onset, ranging from 28 to 31 years, and similar phenotypes observed among family members, we speculated that their ALS was of a monogenic form.

Whole‐exome sequencing was conducted on three affected and two unaffected family members to comprehensively identify rare variants. We aimed to identify variants that were consistently present in all affected individuals but absent in the unaffected members. Given the unique clinical presentation of early‐onset ALS in this family and the expected extreme rarity of the disease, we further narrowed our analysis by focusing on variants not found in public population databases or our in‐house healthy control exome database. Using these stringent selection criteria, we identified three variants in three genes as prime candidates associated with the disease. Ultimately, a novel variant (c.212C > T, p.Ala71Val) in *SPTLC2*, with a combined annotation‐dependent depletion (CADD) score of 23.0, emerged as the only variant exceeding the threshold of 20, indicating a substantial functional impact based on in silico predictions (Figs. S1 and S2). Upon examination of the rare exome variant ensemble learner (REVEL) scores, the *SPTLC2* variant emerged as the most likely pathogenic variant among our candidate variants (Table S1). This variant substitutes a valine residue in place of an evolutionarily conserved alanine residue (Fig. 1B,C).

### Identification of a de novo variant in *SPTLC2* in a patient with early‐onset ALS

Our genetic analysis of Pedigree 2 provided further evidence supporting the pathogenicity of *SPTLC2*. In our investigation of a family with early‐onset SALS, we obtained DNA samples from both the affected individual (proband) and the unaffected parents, facilitating a trio analysis. We confirmed paternity and maternity using identity‐by‐descent analysis based on the trio's whole‐exome sequence data (Methods). This trio analysis yielded three candidate de novo variants with amino acid substitutions, each having an MAF <0.01 (see Methods and Table S2). Among these, we identified a previously unknown heterozygous variant in *SPTLC2* (NM_004863: c.203 T>G, p.Met68Arg) (pedigree 2; Fig. 1A and Fig. S3). This variant replaces an evolutionarily conserved methionine residue with an arginine residue (Fig. 1B,C). Apart from the *SPTLC2* variant, the remaining candidate de novo variants were found in public population databases (gnomAD and ToMMo [jMorp, 38KJPN]) and lacked any reported association with neurological diseases. Therefore, our trio whole‐exome analysis robustly indicates that the novel missense variant (c.203 T>G, p.Met68Arg) in *SPTLC2* is a causal factor for early‐onset ALS.

### *SPTLC2* variants and protein structure analysis

**Figure 2:**
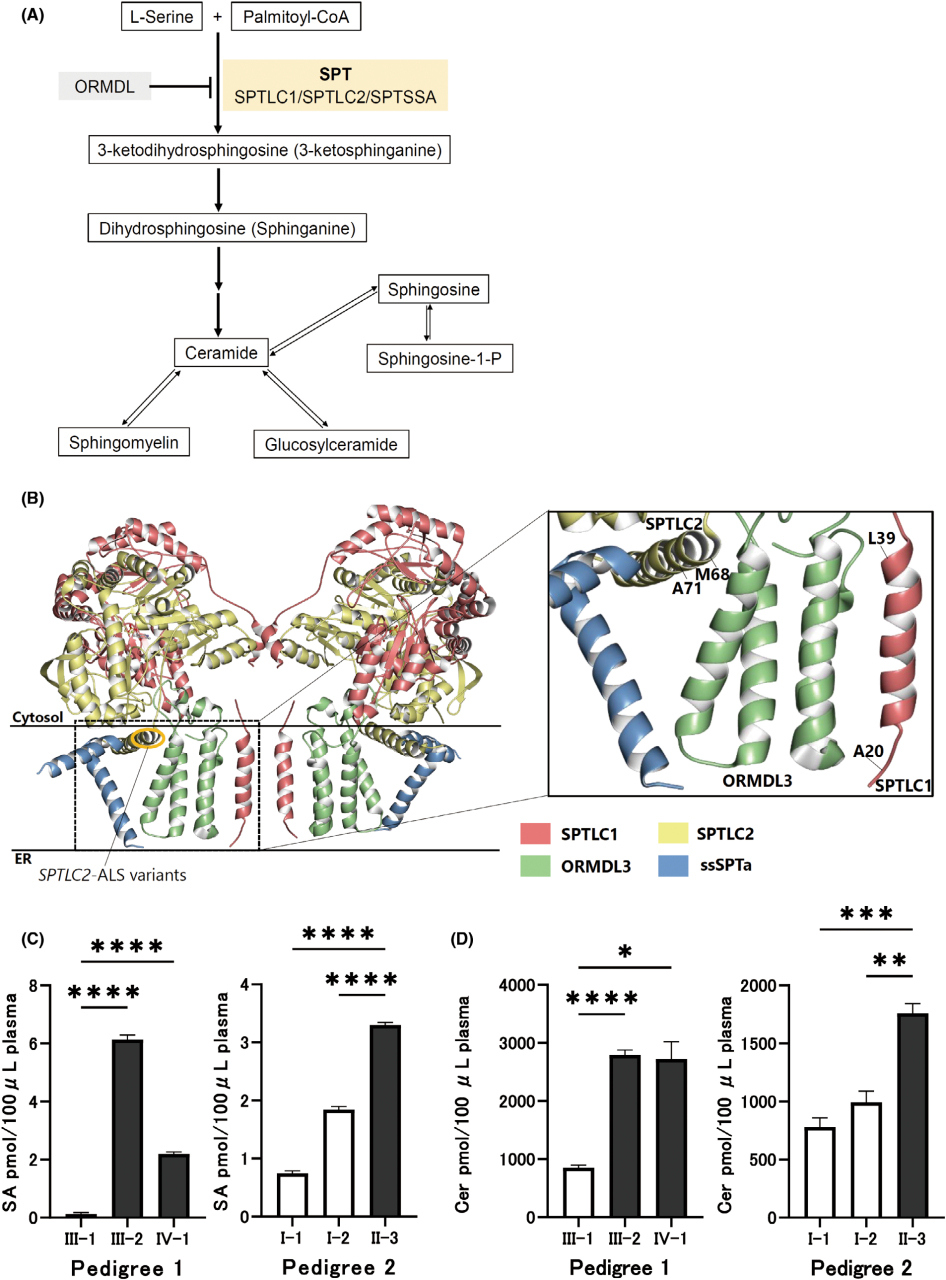
Structure of the SPT/ORMDL3/ssSPTa complex by cryo‐electron microscopy and elevated sphingolipid products in *SPTLC2*‐associated ALS. (A) The canonical sphingolipid metabolic pathway is outlined, highlighting SPT as the initial, rate‐limiting step in sphingolipid biosynthesis. This process culminates in the synthesis of ceramide, which serves as a crucial hub for downstream metabolite production. SPT activity is subject to feedback inhibition by ORMDL proteins. (B) The complex protein structure is displayed as a ribbon representation, with distinct colors designating individual protein entities. This structure was derived from PDBj entry 6M4N and was visualized using the Molmil molecular viewer. *SPTLC2* variants associated with early‐onset ALS are located in the membrane‐associated region of SPTLC2. As shown in the detailed view, ALS‐associated *SPTLC2* variants are located near the ORMDL3‐interacting membrane‐associated region of SPTLC2. The *SPTLC1*‐ALS variants, as exemplified here, were also found near the ORMDL3‐interacting transmembrane region of SPTLC1. ER, endoplasmic reticulum. (C and D) Sphingolipid analyses of plasma samples from affected and unaffected individuals in the two early‐onset ALS families were performed using liquid chromatography‐electrospray ionization‐tandem mass spectrometry (LC‐ESI‐MS/MS). Patients with ALS exhibited significantly elevated levels of base‐form sphingolipid species, such as sphinganine (SA), compared with those in healthy family members (C). Notably, the ceramide (Cer) levels, which comprise fatty acids of various chain lengths, were also significantly higher in patients with ALS than those in healthy family members (D). Measurements were made three to four times for each condition and variant, and statistical comparisons were made within each family. Welch's analysis of variance (ANOVA) was performed, followed by Dunnett's multiple comparison tests to identify significant differences. Error bars represent the standard error of the mean (SEM). An adjusted *p*‐value <0.05 was considered statistically significant. In the graphs, *p < 0.05, **p < 0.01, ***p < 0.001, and ****p < 0.0001.

SPTLC2, in concert with SPTLC1 and the serine palmitoyltransferase small subunit (ssSPT), forms an integral component of the multi‐subunit SPT complex, the principal enzyme facilitating the initial, rate‐limiting step in de novo sphingolipid biosynthesis. This process generates long‐chain base compounds by condensing an amino acid, typically serine, with activated acyl‐CoA, primarily palmitoyl‐CoA.^21^, ^22^ A series of reactions subsequently form ceramide, a critical hub to produce downstream sphingolipids (Fig. 2A).^23^

Pathogenic variants in the cytoplasmic domains of SPTLC1 and SPTLC2 can cause hereditary sensory and autonomic neuropathy type 1 (HSAN1) and macular telangiectasia type 2 by abnormally increasing deoxy‐sphingolipid levels.^24^, ^25^, ^26^, ^27^, ^28^, ^29^ SPT preferentially metabolizes alanine or glycine instead of serine to generate toxic deoxy‐sphingolipids under these conditions. These toxic compounds cannot be metabolized or degraded through canonical sphingolipid catabolism, leading to their cellular accumulation and appearance in the plasma (Fig. S4).^30^ While deoxy‐sphingolipid accumulation in HSAN1 primarily affects peripheral nerves, central nervous system (CNS) symptoms are rarely observed in patients with this condition, suggesting minimal CNS involvement.

The recent elucidation of the structure of the human SPT/ORMDL3 complex by cryo‐electron microscopy revealed that the SPT multi‐subunit complex comprises SPTLC1, SPTLC2, and ssSPT (Fig. 2B).^19^, ^31^ Contrasting with previously reported HSAN1‐causing variants within the cytoplasmic domain of SPTLC2,^26^, ^29^, ^32^, ^33^, ^34^, ^35^ our study reveals that the newly identified variants in *SPTLC2*, p.Met68Arg and p.Ala71Val, are located within the membrane‐associated region (Fig. 1C). This localization is similar to that observed in *SPTLC1*, as illustrated in Figure S5.^24^, ^25^, ^27^, ^28^, ^29^ Notably, these *SPTLC2* variants and ALS‐causing *SPTLC1* variants,^10^, ^11^, ^19^ both affecting amino acids within the membrane‐associated region, share the characteristic of being adjacent to ORMDL3, a regulator within this complex, thereby highlighting an intriguing parallel.

The SPT enzymatic activity is modulated by its downstream products, with ceramide synthesis and ORMDL proteins playing essential roles in homeostatic inhibition.^36^, ^37^ Among ORMDLs, ORMDL3 deficiency in mice resulted in elevated sphingolipid levels in the central nervous system compared to its analogs Ormdl1 and Ormdl2.^38^

*SPTLC1* variants associated with early‐onset ALS are postulated to enhance SPT activity by interfering with the binding of mutant SPT to ORMDL3, promoting the overproduction of sphingolipids, thereby leading to ALS manifestation.^10^, ^39^ Similarly, the *SPTLC2* variants identified in our study, located within the membrane‐associated region adjacent to ORMDL3, indicated that these variants alter the SPT–ORMDL3 interaction, leading to the disruption of sphingolipid homeostasis.

### *SPTLC2* variants and lipid analysis

We investigated this further by analyzing sphingolipids in plasma samples from affected and unaffected members of two families with early‐onset ALS employing LC‐ESI‐MS/MS as described in Supplementary Methods.^20^ For Pedigree 1, which consisted of patients with early‐onset FALS harboring the *SPTLC2* p.Ala71Val variant, samples from two affected and one unaffected individual were analyzed. Concurrently, in Pedigree 2, which involved a patient with early‐onset SALS with the *SPTLC2* p.Met68Arg de novo variant, samples from the affected individual and both unaffected parents were analyzed.

Notably, significantly elevated levels of base‐form species of sphingolipids, such as sphinganine (SA), were found in two patients (III‐2 and IV‐1) compared to the healthy family member (III‐1) in Pedigree 1, and in the patient (II‐3) compared to the healthy family members (I‐1 and I‐2) in Pedigree 2 (Fig. 2C). However, the levels of 1‐deoxy‐sphinganine (doxSA), 1‐deoxymethyl‐sphinganine (doxmeSA), 1‐deoxy‐sphingosine (doxSO), and 1‐deoxymethyl‐sphingosine (doxmeSO) remained unchanged in patients with ALS and healthy family members within each family (Fig. S4). In addition, upon investigating fatty acid‐acylated forms of sphingolipids, the levels of ceramide (Cer), composed of fatty acids of various chain lengths, were significantly higher in patients with ALS than those in their respective healthy family members (Fig. 2D and Table S3). Consequently, we demonstrated increased free sphinganine and circulating ceramide levels in the affected individuals, which aligns with the pathophysiological pattern observed in patients with early‐onset ALS carrying pathogenic *SPTLC1* variants.^10^

### Clinical profile of patients with early‐onset ALS carrying novel *SPTLC2* variants

The Table 1 summarizes the clinical characteristics of patients with early‐onset ALS carrying the newly identified *SPTLC2* variants. Each patient displayed both lower and upper motor neuron signs across various spinal cord and brainstem regions, leading to a diagnosis of either possible or probable ALS.^13^ Notably, all patients manifested cognitive dysfunction, presenting with a range of symptoms, including behavioral disinhibition, apathy, increased stereotypic behaviors, and a decline in executive functioning. Cognitive dysfunction is particularly evident in patients with FALS from the early stages of the disease. As the disease progressed, all of the patients exhibited signs of FTD. Remarkably, Patient 2 was diagnosed with ALS‐FTD at the initial evaluation. Tongue atrophy was a common early symptom in all the patients, whereas sensory deficits were absent throughout the study. Some patients experienced emaciation and respiratory disturbances. Ophthalmological examinations showed that none of our SPTLC2‐ALS patients exhibited macular telangiectasia or other notable retinal abnormalities. More detailed clinical information on these patients is available in the Supplementary Methods.

**Table 1:** Clinical characteristics of the patients with early‐onset ALS carrying *SPTLC2* variants.

Pedigree	1	1	1	2
Patient	III‐2 (Patient 1)	III‐3 (Patient 2)	IV‐1 (Patient 3)	II‐3 (Patient 4)
Variant^a^	p.Ala71Val	p.Ala71Val	p.Ala71Val	p.Met68Arg
Inheritance	AD	AD	AD	*De novo*
Age at onset	28	31	31	22
Initial symptoms	Cognitive impairment	Cognitive impairment	Cognitive impairment	Abnormal gait (LE)
Age at evaluation	35	42	34	30
Walking	Abnormal (ataxic and spastic)	Abnormal (ataxic and spastic)	Abnormal (ataxic and spastic)	Nonambulatory (ataxic and spastic)
Atrophy	Global	NI	Global	UE predominant
Weakness	Generalized	NI	Generalized	UE predominant
Reflexes	Hyporeflexia, Achilles tendon brisk	Hyperreflexia in UE and LE	Hyperreflexia in UE and LE	Hyporeflexia in UE, hyperreflexia in LE
Tongue	Wasted, fasciculations	Wasted, fasciculations	Wasted, fasciculations	Wasted, fasciculations
Respiratory insufficiency^b^	++	−	−	++
Exam: LMN sign	Cr, C, T, L	Cr, C	Cr, C, L	Cr, C
Exam: UMN sign	L	Cr, C, L	L	Cr, C, T, L
Cognitive impairment^c^	+; FTD suspected	+; FTD	+; FTD suspected	+; FTD suspected
Brain MRI	T2‐weighted low‐intensity in IGP	Diffuse frontotemporal atrophy	No significant abnormalities	Frontal lobe atrophy
Sensory exam^d^	Normal	Normal	Normal	Normal
*Neurophysiology*				
Sensory NCS	Normal	Normal	Normal	Normal
Motor NCS: CMAP^e^	Decreased amplitudes	Decreased amplitudes	Decreased amplitudes	Decreased amplitudes
EMG	Acute and chronic denervation	Acute and chronic denervation	Acute and chronic denervation	Acute and chronic denervation
Additional features	Bilateral cataracts			Scoliosis

AD, autosomal dominant; C, cervical region; CMAP, compound muscle action potential; Cr, cranial region; EMG, electromyography; FTD, frontotemporal dementia; IGP, internal globus pallidus; L, lumbar region; LE, lower extremity; LMN, lower motor neuron signs; NCS, nerve conduction studies; NI, not indicated; T, thoracic region; UE, upper extremity; UMN, upper motor neuron signs.

^a^ RefSeq ID: NP_004854.

^b^ ++, use of noninvasive or invasive (i.e., tracheostomy) ventilatory support.

^c^ Patients 1, 2, and 4 demonstrated cognitive dysfunction suggestive of FTD during their clinical course, leading to the suspicion of concomitant FTD.

^d^ Bedside sensory tests for pinprick, temperature, vibration, and proprioception.

^e^ Distal latency and nerve conduction velocity tests within normal limits.

### Mutational analysis of *SPTLC2* in patients with adult‐onset ALS and control subjects

In our study of 62 probands with early‐onset ALS, novel *SPTLC2* variants were identified in two probands: one from a FALS family and the other in a SALS patient who presented de novo (2 out of 62, 3.2%). Additionally, focusing on the 37 probands without identified pathogenic variants in ALS‐causing genes, the frequency of *SPTLC2* variants is 5.4% (2 out of 37). At the time of genetic analysis, none of the remaining patients with early‐onset ALS were diagnosed with ALS‐FTD.

We extended our analysis to patients with adult‐onset ALS, excluding those with early‐onset ALS (Table S4). In this group, 2 familial and 11 sporadic patients with ALS were diagnosed with ALS‐FTD. No *SPTLC2* variants were detected in the patients with FALS. Nonetheless, *SPTLC2* variants were detected in 0.5% of the patients with SALS and 0.26% of healthy controls (Table S5). All identified variants were located at sites distinct from the membrane‐associated region. The pathogenicity of these variants in patients with SALS could not be assessed because of the unavailability of plasma samples from the affected individuals and DNA samples from their unaffected family members.

## Discussion

In this study, we identified previously unknown *SPTLC2* variants in patients with early‐onset ALS. These variants are located within the membrane‐associated region adjacent to ORMDL3. Based on clinical presentation, they indicate distinct etiologies compared to those of HSAN patients. Our protein structure and lipid analyses demonstrate that these variants disrupt SPT‐ORMDL3 binding, leading to dysregulated sphingolipid homeostasis and promoting excessive sphingolipid synthesis. These findings provide compelling evidence for *SPTLC2* variants linked to early‐onset ALS with FTD.

Our study underscores the diverse impacts of *SPTLC2* variants, particularly in the membrane‐associated region, which are associated with ALS, mirroring similar findings in *SPTLC1*.^10^, ^11^ Variants in the membrane‐associated region lead to increased production of typical sphingolipids such as ceramide, in contrast to the deoxy‐sphingolipid abnormalities resulting from variants in other regions of the *SPTLC1/2* genes, often linked to HSAN1.^10^, ^30^ This distinction in variant locations within *SPTLC2* is crucial for understanding the broad spectrum of clinical manifestations and associated lipid metabolic pathways (Fig. S4). Our insights not only expand our comprehension of ALS's genetic spectrum but also highlight how specific genetic variations in the SPT subunit contribute uniquely to the pathogenesis of distinct neurological conditions.

Sphingolipids, which are vital components of human cellular membranes, are directly affected by alterations in SPT activity. In our study, significantly elevated plasma ceramide levels consisting of sphingosine attached to a fatty acid tail were detected in patients with *SPTLC2*‐ALS compared to those in healthy individuals. Previous studies have reported elevated ceramide levels in the spinal cords of *SOD1*‐G93A mice, as well as in the plasma, serum, and spinal cords of patients with SALS, implicating oxidative stress‐induced sphingolipid synthesis.^40^, ^41^, ^42^, ^43^ Moreover, presymptomatic ALS mice demonstrated increased ceramide levels, suggesting a causative role for ALS‐associated motor neuron degeneration.^40^ It has been reported that ceramide triggers apoptotic cell death in cortical and motor neurons.^44^, ^45^, ^46^, ^47^ These findings indicate that the overproduction and consequent accumulation of sphingolipids such as ceramide contribute to ALS pathogenesis, highlighting ceramides as promising ALS biomarkers and therapeutic targets.

The function of SPT has been associated with hereditary spastic paraplegia (HSP). Recent research has revealed variants in the SPTSSA transmembrane region near ORMDL3, leading to complex HSP through ORMDL‐mediated dysregulation of sphingolipid synthesis.^48^ In our ALS case series, we did not detect any rare variants in *SPTSSA*, indicating that the genetic contributions to ALS may be different from those in HSP. This underscores the need for further investigation into a broader range of cases, especially in HSP, to better understand the role of SPT and related genes in different neurodegenerative conditions.

Therapeutic strategies are continually evolving in the quest to manage and treat complex neurological disorders like ALS. Recent studies proposing oral L‐serine therapy for HSAN^49^, ^50^ have highlighted its potential applicability for patients with early‐onset ALS carrying *SPTLC1* or *SPTLC2* variants. The underlying pathogenesis of HSAN is thought to stem from the heightened vulnerability of sensory nerves to deoxy‐sphingolipids, compared to motor nerves.^30^ Increasing the serine/alanine ratio may promote mutant SPT binding to serine, reduce deoxy‐sphingolipid synthesis, and potentially decelerate disease progression. However, our lipid analysis has shown that in the patients with *SPTLC2*‐ALS, the increase in ceramide levels is more pronounced than the accumulation of deoxy‐sphingolipids, which was attributed to heightened SPT activity. Such findings raise concerns that oral L‐serine therapy could inadvertently exacerbate the symptoms in these individuals. Furthermore, it is imperative to recognize the findings from recent studies on SPTLC1‐ALS, where lipid analysis similar to our study revealed that L‐serine supplementation, rather than providing therapeutic benefits, may exacerbate the biochemical phenotype in these patients.^10^ In light of these findings, future therapeutic strategies should prioritize controlling sphingolipid synthesis and preventing its accumulation to manage *SPTLC2*‐ALS effectively.

A limitation of our study is the absence of autopsy data on the deceased individuals from Pedigree 1, which prevents us from exploring neuropathological features associated with SPTLC2‐ALS. Additionally, the lack of longitudinal ALSFRS‐R assessments in our study precludes a comprehensive analysis of disease progression in patients with SPTLC2‐ALS. Furthermore, a recent study in a cohort of 2011 ALS patients from the Chinese population identified 16 variants outside the membrane‐associated region of *SPTLC2*.^51^ This discovery highlights the genetic diversity within *SPTLC2* and underscores the necessity for further research to explore the broader implications of these variants in ALS, especially those located outside the membrane‐associated region.

Very recently, concurrent with our study, there have been reports linking *SPTLC2* variants with juvenile ALS. Notably, the Met68Arg variant, which we also identified in a de novo SALS patient, was reported in two additional de novo patients.^52^ Moreover, the Glu260Lys variant was observed in six de novo patients.^53^ These recent findings predominantly featured juvenile ALS, highlighting the critical role of specific *SPTLC2* variants in ALS pathogenesis. Differing from these reports, our research identified the Ala71Val variant in three affected patients within a FALS pedigree, which exhibited an ALS‐FTD clinical spectrum. This finding provides unique insights into the diverse clinical manifestations associated with *SPTLC2* variants.

In conclusion, our study unveiled previously unknown, dominantly‐acting *SPTLC2* variants in early‐onset ALS with FTD. Compelling genetic findings, coupled with the significant elevation in plasma ceramide levels, established a pivotal link between dysregulated sphingolipid metabolism and ALS pathogenesis. These observations not only broaden our understanding of genetic diversity in ALS but also underscore the distinct roles of gene defects in the membrane‐associated region and other areas within the same SPT subunit, which contribute differently to the disease pathogenesis. Furthermore, our findings highlight the potential of sphingolipids as biomarkers and therapeutic targets for ALS, marking a significant step toward precision medicine for this devastating disease.

## Author Contributions

H.N., H.I., K.E., T.Y., S.T., and T.T. contributed to the conception and design of the study; H.N., H.I., K.E., J.M., W.S., P.G., N.S., Y.M., Y.K., H.H., T.O., T.I., I.Y., Y.T., Y.S., T.M., J.G., K.K., Y.T., S.M., T.Y., S.T., and T.T. contributed to the acquisition and analysis of data; H.N., H.I., K.E., J.M., W.S., P.G., N.S., Y.M., Y.K., H.H., T.O., T.I., I.Y., Y.T., Y.S., T.M., J.G., K.K., Y.T., S.M., T.Y., S.T., and T.T. contributed to drafting the text or preparing the figures.

## Conflict of Interest

The authors have no conflict of interest to declare.

## Supporting information


Appendix S1.

## Data Availability

The data that support the findings of this study are available on request from the corresponding author.
