# Supplementary material for: SPTLC2 variants are associated with early‐onset ALS and FTD due to aberrant sphingolipid synthesis
Source: Ann Clin Transl Neurol. 2024 Feb 5;11(4):946–57. doi: 10.1002/acn3.52013 (PMC11021611; doi:10.1002/acn3.52013)
Supplement: Supplementary file 1 — Appendix S1. [file ACN3-11-946-s001.pdf]

Supplementary Materials

Supplementary Methods.

Supplementary Figure 1. Variant filtering strategy based on whole-exome sequence analysis

Supplementary Figure 2. *SPTLC2* p.Ala71Val variant associated with early-onset ALS

Supplementary Figure 3. *SPTLC2* p.Met68Arg variant associated with early-onset ALS

Supplementary Figure 4. Divergence of SPT enzyme pathways in HSAN1 and ALS

Supplementary Figure 5. Schematic representation of the SPTLC1 protein

Supplementary Table 1. Candidate variants identified in pedigree 1 through the filtering steps

Supplementary Table 2. *De novo* variants identified in pedigree 2 through the filtering steps of *de novo* analysis

Supplementary Table 3. Levels of sphingolipids in the plasma from the ALS-affected patients and controls in the pedigrees

Supplementary Table 4. Demographic and clinical features of patients with adult-onset ALS and controls screened for *SPTLC2* variants

Supplementary Table 5. *SPTLC2* variants identified in patients with adult-onset ALS and controls

Supplementary Methods.

A detailed description of patient 1

Patient 1, a 35-year-old female (III-2 in pedigree 1), presented to our hospital with the chief complaint of muscle atrophy in her extremities. She had a family history of ALS, and her father was clinically diagnosed with ALS and died at 42. The patient had no perinatal or subsequent developmental abnormalities, and her medical history was unremarkable.

She exhibited noticeable forgetfulness, often repeated herself, and displayed immediate short-term memory loss from the age of 27 years. She sometimes purchased the same item multiple times while shopping. At the age of 30 years, she struggled to maintain the same pace as others during walking and became short of breath when walking briskly. People around her noticed muscle atrophy in her extremities. Over time, she started stumbling and falling, even on low steps, and her gait became unstable. At the age of 34 years, she became less attentive to her appearance, often repeating herself and neglecting household chores. The patient was hospitalized at the age of 35 years.

The patient, on examination, displayed emaciation (height, 150 cm; weight, 26.5 kg; body mass index (BMI), 11.8). Bilateral cataracts were observed, with no evidence of retinitis pigmentosa or papillary atrophy. In terms of cranial nerves, a left eyelash sign was noted. The pharyngeal reflex was positive. The patient exhibited hoarseness and dysphagia. Dysarthria was present, along with weak tongue protrusion. Tongue atrophy and fasciculation were also observed. Regarding the motor system, generalized atrophy of the extremity muscles was observed, yet muscle strength was relatively preserved at 3–4/5 on the Medical Research Council (MRC) scale. Muscle weakness was more pronounced in distal muscles, particularly the small hand muscles, exhibiting a "split hand" pattern often associated with ALS. This pattern involves the atrophy of the thenar and first dorsal interosseous (FDI) muscles, whereas the hypothenar muscles remained relatively preserved. Weakness of the tibialis anterior (TA) muscle was also observed. The muscle tone was decreased in the upper extremities but was mildly spastic in the lower extremities. Bilateral ATRs were hyperactive. The right PTR was normal; however, the other reflexes were absent or decreased. The Babinski reflex was negative, while bilateral ankle clonus was present. No obvious abnormalities in the tactile, thermal, pain, vibratory, or positional sensations were observed in the sensory system. Her gait was ataxic and spastic with foot drops. Romberg's sign was negative.

Cognitive assessment using the Wechsler Adult Intelligence Scale-Revised (WAIS-R) revealed a verbal intelligence quotient (V-IQ) of 57, a performance IQ (P-IQ) that could not be scaled, and a total IQ (T-IQ) of 45, indicating impaired cognitive function. Computed tomography (CT) of the head revealed bilateral calcification of the globus pallidus and mild cortical atrophy. Magnetic resonance imaging (MRI) of the head revealed low-intensity T2-weighted images of the internal segment of the globus pallidus, with no brainstem or cerebellar atrophy. Brain single-photon emission computed tomography (SPECT) showed no apparent hypoperfusion. Nerve conduction studies (NCSs) revealed a reduced amplitude of the compound muscle action potential (CMAP) and the absence of the F wave in the right median nerve, while sensory conduction studies (SCSs) were normal. Auditory brainstem response (ABR) and somatosensory-evoked potential (SEP) results were within normal limits. Needle electromyography (nEMG) was performed in the right sternocleidomastoid (SCM), deltoid, biceps, triceps, extensor carpi radialis (ECR), FDI, quadriceps femoris (QF), hamstrings, and TA muscles, showing large motor unit potentials (MUPs) in all the examined muscles. Spontaneous high-frequency discharges (bizarre high-frequency potentials) were observed in several muscles.

The histological examination of the muscle biopsy specimen revealed severe fascicular atrophy, increased connective tissue, and fat infiltration, as observed on hematoxylin and eosin staining. The remaining muscle fibers exhibited pseudomyopathic changes with no evidence of necrosis, regeneration, or inflammation. NADH staining showed a predominance of type 2 fibers, which were lightly stained, and scattered lobulated fibers. PAS staining revealed a predominance of relatively good stained type 2 fibers. ATPase staining revealed fiber-type grouping, with type 2 only in the fasciculus, and type 2 and type 1 fibers divided into two separate fasciculi.

The patient underwent a tracheotomy at the age of 35 years and began using a button tracheostomy retention tube; then, she became ventilator-dependent at the age of 47 years.

A detailed description of patient 2

Patient 2 was a 42-year-old male (III-3 in pedigree 1) who presented with the chief complaint of cognitive decline. The patient had no perinatal or developmental abnormalities and his medical history was unremarkable.

At the age of 31 years, he began to struggle with his work at an electronics factory, where he had been employed since he was 18 years old. His work performance declined, and tasks took longer to complete. He eventually became unable to perform his duties and colleagues commented on his growing laziness. At the age of 40 years, he lost his job and subsequently another, finally ceasing to work entirely. He showed little awareness of his condition, spent most of his time at home, and left his house only for occasional shopping. Concerns from his family regarding his cognitive decline, along with a gradual progression of spasticity and muscle weakness in his extremities, led to his hospitalization at the age of 42 years.

On neurological examination of the cranial nerves, gaze-evoked nystagmus in both directions, dysarthria, and mild atrophy and fasciculation of the tongue were observed. In the motor system, there was no obvious muscle weakness in the neck or extremities; however, fasciculation was observed in the extremities. Deep tendon reflexes were enhanced in the left upper extremity, and PTR and ATR were enhanced in both lower extremities. A positive jaw jerk reflex, bilateral positive Hoffmann and Tromner reflexes, positive Babinski reflex on the right side, and bilateral positive palmomental reflexes were observed. Sensory examination revealed no abnormalities in the tactile, thermal, pain, vibratory, or positional sensations. His gait was ataxic with a waddling pattern, and Romberg's sign was negative. No autonomic symptoms were present.

The patient obtained a cognitive function assessment score of 23/30 on the Mini-Mental State Examination (MMSE), tending to repeat words and unable to perform simple hand imitations. The WAIS-R revealed a V-IQ of 63, a P-IQ of 51, and a T-IQ of 53, indicating diminished cognitive function. Brain SPECT demonstrated markedly reduced blood flow in the frontal and temporal regions. Head MRI revealed diffuse frontotemporal brain atrophy and mild enlargement of the fourth ventricle. Motor conduction studies (MCSs) showed impairment of the left and right ulnar nerves in the elbows, whereas SCSs were normal. nEMG performed on the tongue, biceps, triceps, QF, TA, and gastrocnemius (GC) muscles revealed neurogenic changes and fibrillation potentials (Fib) throughout, absence of fasciculation, and repetitive discharge in the biceps. Neurogenic changes were present in the bulbar region and upper and lower extremities.

Head MRI, brain SPECT, and neuropsychological testing showed frontotemporal lobe dysfunction. The patient was considered to have ALS-frontotemporal dementia (ALS-

FTD) spectrum based on these findings.

A detailed description of patient 3

Patient 3 was a 34-year-old female (IV-1 in pedigree 1) who presented to the hospital with the chief complaint of cognitive decline. She had no perinatal or developmental abnormalities, and her medical history was unremarkable.

At the age of 31 years, she was assigned a new role at a car-cleaning company, where she had been employed for approximately 10 years. She found adapting to her new tasks challenging, which led her to take a year off work. At the age of 33 years, her performance at work gradually declined, prompting her to switch jobs. Her father noticed a remarkable decline in her memory and attributed her poor job performance to this cognitive decline. She could slowly perform previously learned tasks but struggled to acquire new ones and showed little self-awareness of her condition. At the age of 34 years, she was admitted to a hospital because her father was concerned about her cognitive decline.

On examination, the patient presented with emaciation (height, 145 cm; weight, 33 kg; BMI, 15.7) and blinked infrequently. Mild atrophy and fasciculation of the tongue were observed in the cranial nerves. In the motor system, diffuse atrophy of the extremity muscles was noted, with muscle strength measuring approximately 3–4/5 on the MRC scale. Muscle tone in the lower extremities was mildly spastic. Deep tendon reflexes were normal in the upper extremities, while PTR and ATR were enhanced in the lower extremities, with no difference between the right and left sides. The Babinski reflex was positive bilaterally. Sensory examination revealed no abnormalities in the tactile, thermal, pain, vibratory, or positional sensations. Her gait was unstable and waddling, and she could not perform a tandem gait. Romberg's sign was negative.

Cognitive function assessment indicated an MMSE score of 23/30, Frontal Assessment Battery (FAB) score of 16/18, and Wechsler Adult Intelligence Scale-Third Edition (WAIS-III) scores of 50 for V-IQ and 57 for T-IQ. nEMG was performed on the biceps, triceps, ECR, FDI, QF, TA, GC, and SCM muscles. A resting discharge was observed only in the GC, an interference pattern was noted only in the biceps, and the other muscles showed poor activation. Chronic neurogenic changes and polyphasic MUPs were observed in all the examined muscles. The NCSs were within the normal range. Head and cervical spine MRI revealed no significant abnormalities. A follow-up cognitive

assessment conducted at the age of 38 years displayed an MMSE score of 22/30 and a FAB score of 9/18, indicating FTD.

A detailed description of patient 4

Patient 4 (II-3 in pedigree 2) was a 30-year-old male initially presenting with muscle atrophy in both hands and a gait disturbance. His perinatal and subsequent developmental stages were normal, and he had a history of undescended testicles that resolved spontaneously.

At the age of 22 years, he began to exhibit lateral flexion of the trunk while walking, flattened palms, and fatigue when carrying heavy loads. His dropped neck and gait disorders gradually progressed, leading to hospitalization for comprehensive examination at the age of 26 years. His family history did not include consanguineous marriage or similar diseases.

Upon admission, the neurological findings revealed dysarthria, a high-arched palate, and atrophy and fasciculation of the tongue. Muscle atrophy was observed in the bilateral dorsal interosseous and adductor pollicis brevis muscles, along with weakness in the cervical muscles and bilateral upper extremities, predominantly in the distal muscles. Contraction fasciculations were present in both upper extremities. Spasticity was observed in the lower extremities. Deep tendon reflexes were decreased in the upper extremities and markedly increased bilaterally in the lower extremities. The jaw jerk reflex was positive, Hoffmann and Tromner reflexes were positive on the left, and the Babinski reflex was extensor on the right. Ankle clonus was sustained bilaterally, with an absent abdominal reflex. Sensory examination revealed normal tactile, temperature, pain, vibration, and positional sensations. The patient's gait was broad-based with a waddling pattern and Romberg's sign was negative.

The nEMG examination showed chronic neurogenic changes in the biceps and FDI muscles, with both active and chronic neurogenic changes in the TA muscle. NCSs revealed decreased CMAP amplitudes in the right median and ulnar motor nerves, whereas SCSs were normal. Head MRI demonstrated frontal lobe atrophy, enlargement of the lateral and fourth ventricles, and mild thinning of the corpus callosum. Brain SPECT and cervical spine MRI revealed no specific findings. Ophthalmological

examination revealed no retinal or optic papillary abnormalities. The initial cognitive assessment at the age of 26 years indicated an MMSE score of 28/30 and a FAB score of 16/18, with no obvious cognitive or frontal lobe deficits.

Follow-up nEMG at the age of 28 years revealed positive sharp waves (PSW) and Fib at rest in the right TA and biceps muscles. The cognitive assessment showed a decline, with an MMSE score of 19/30 and a FAB score of 11/18 at the age of 28 years. At the age of 30 years, the patient was hospitalized for CO_2 narcosis due to respiratory muscle fatigue, leading to subsequent intubation and mechanical ventilation.

Sphingolipid measurements

The internal standard materials, purchased from Avanti Polar Lipids (Alabaster, AL, USA) for sphingolipid analysis, comprised the internal standard mixture (ceramide/sphingoid internal standard mixture I), D-erythro-sphinganine-d7, D-erythro-sphingosine-d7, D-erythro-sphingosine-d7-1-phosphate, 1-deoxy-sphinganine-d3 (m18:0), 1-deoxymethyl-sphinganine-d5 (m17:0), N-lauroyl-dihydroceramide (C12DHCer), N-C12–1-deoxy-dihydroceramide (C12doxDHCer), N-C12–1-deoxy-ceramide (C12doxCer), N-C12-1-deoxymethyl-dihydroceramide (C12doxmeDHCer), and N-C12-1-deoxymethyl-ceramide (C12doxmeCer).

For the lipid extraction and analysis, 300 µl of cold water was mixed with 100 µl of each plasma sample. After adding 1.5 ml of chloroform/methanol (1:2, v/v), the homogenate was added to the internal standard mixture and incubated at 48 °C overnight. After adding 150 µl of 1 M potassium hydroxide in methanol, the mixture was incubated for 2 h at 37 °C, and then neutralized by adding glacial acetic acid. For the analysis of base-form sphingolipids (sphingoid base: sphinganine [SA], sphingosine [SO], sphingosine-1-phosphate [S1P], 1-deoxy-sphinganine [doxSA], 1-deoxy-sphingosine [doxSO], 1-deoxymethyl-sphinganine [doxmeSA], and 1-deoxymethyl-sphingosine [doxmeSO], Supplementary Fig. 4), samples were centrifuged at 1,500 × *g* for 10 min to obtain the supernatant. The residue was re-extracted with 1 ml of chloroform/methanol (2:1, v/v) and centrifuged at 1,500 × *g* for 10 min. Both supernatants were then combined for further analysis. For the analysis of fatty-acid-acylated-form sphingolipids (dihydroceramide [DHCer], ceramide [Cer], 1-deoxy-dihydroceramide [doxDHCer], 1-deoxy-ceramide [doxCer], 1-deoxymethyl-dihydroceramide [doxmeDHCer], 1-deoxymethyl-ceramide

[doxmeCer], lactosylceramide [LacCer], hexosylceramide [HexCer] and sphingomyelin [SM], Supplementary Fig. 4), 1 ml of chloroform and 2 ml of water were added to the samples; the samples were vortexed well and centrifuged at 1,500 × *g* rpm for 10 min to obtain a two-phase system, the aqueous top phase and the organic bottom phase, from which the lipids were extracted. The top phase was re-extracted with 1 ml of chloroform and centrifuged at 1,500 × *g* for 10 min, after which the organic phases were combined. These lipid extracts were dried under an N_2 stream and stored at -20 °C until further use. The dried residue was dissolved in the mobile phase solvent immediately before LC-ESI-MS/MS analysis using the Nexera X2 HPLC system (Shimadzu, Kyoto, Japan) and QTRAP 4500 (AB SCIEX, Framingham, MA, USA).

Data and materials availability

dbSNP (NCBI single nucleotide polymorphism database; https://www.ncbi.nlm.nih.gov/snp/), gnomAD (The Genome Aggregation Database; https://gnomad.broadinstitute.org/), ToMMo (Tohoku Medical Megabank Organization, jMorp, Japanese Multi Omics Reference Panel, 38KJPN; https://jmorp.megabank.tohoku.ac.jp), Protein Data Bank Japan (PDBj; https://pdbj.org), and PLINK 1.90 (https://www.cog-genomics.org/plink/1.9/).

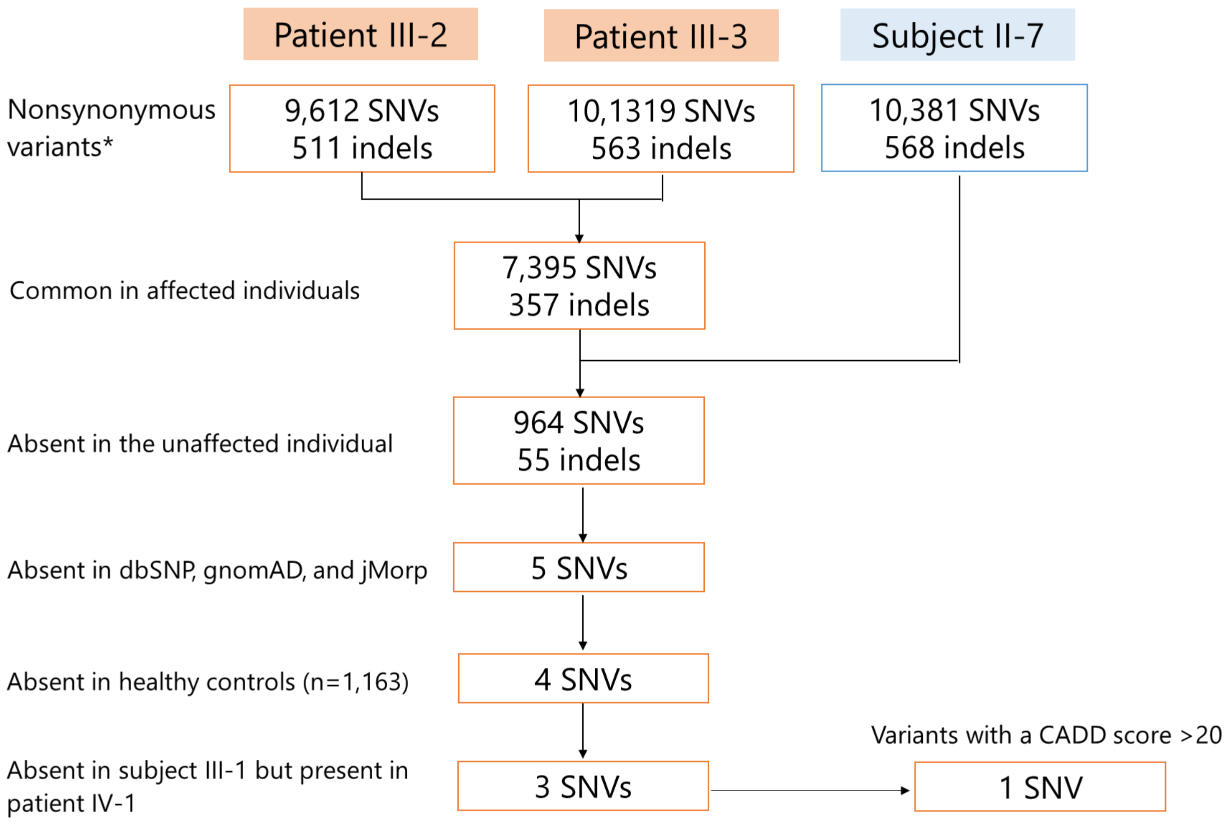

Supplementary Figure 1. Variant filtering strategy based on whole-exome sequence analysis

The figure illustrates the variant filtering strategy for pedigree 1 based on whole-exome sequence analysis data and *in silico* predictions.

*Variants with a quality score <20 or read depth <10 were excluded. The referenced databases, accessed in October 2022, include dbSNP (The Single Nucleotide Polymorphism Database), gnomAD (The Genome Aggregation Database; https://gnomad.broadinstitute.org/), and jMorp (Japanese Multi Omics Reference Panel; https://jmorp.megabank.tohoku.ac.jp; 38KJPN).

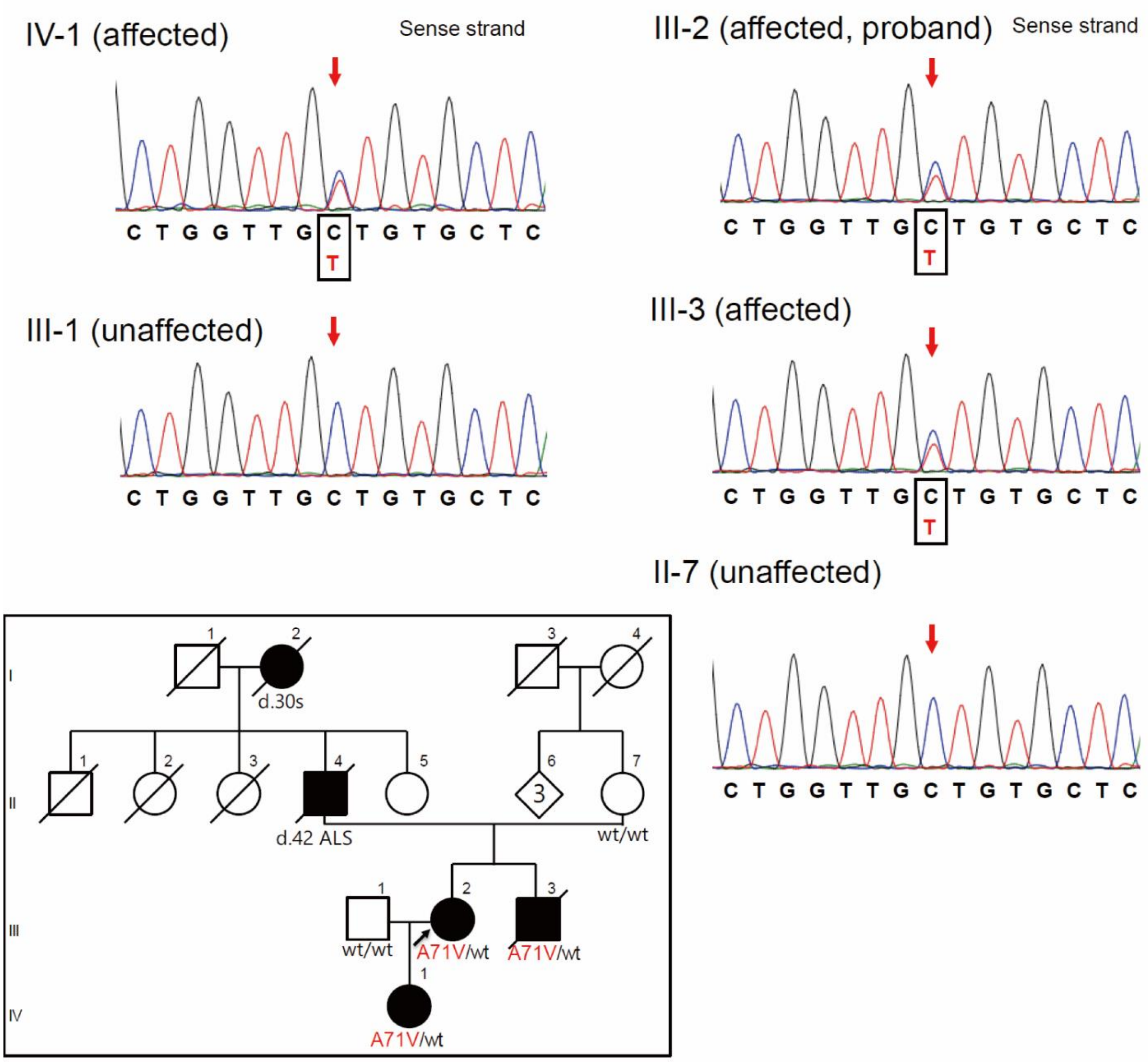

Supplementary Figure 2. *SPTLC2* p.Ala71Val variant associated with early-onset ALS

The pedigree chart shows the presence or absence of the c.212C>T (p.Ala71Val) variant of *SPTLC2*, as confirmed by direct nucleotide sequence analysis. Electropherograms of the sense strand revealed a heterozygous *SPTLC2* variant in three affected individuals (III-2, III-3, and IV-1) and its absence in two unaffected individuals (II-7 and III-1).

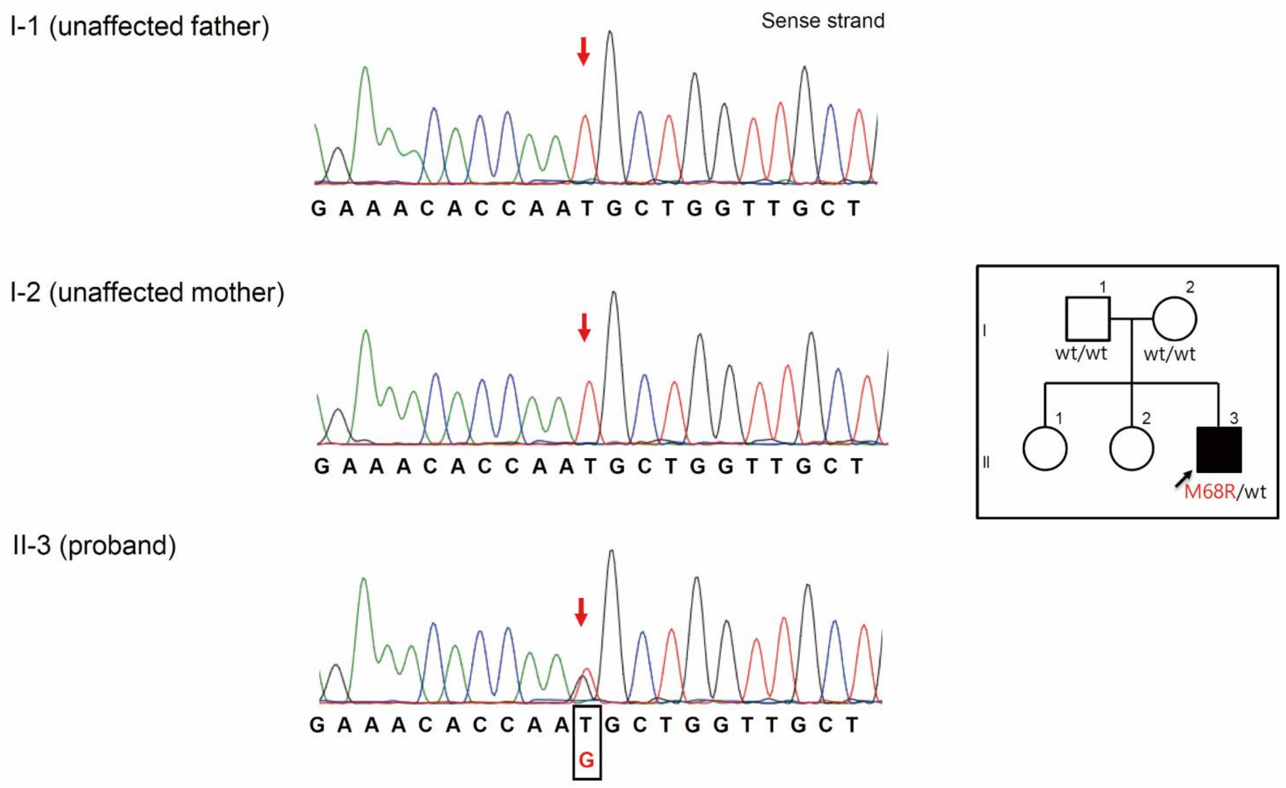

Supplementary Figure 3. *SPTLC2* p.Met68Arg variant associated with early-onset ALS

The pedigree chart depicts the presence or absence of the c.203T>G (p.Met68Arg) variant of *SPTLC2*, which was validated by direct nucleotide sequence analysis. Electropherograms of the sense strand demonstrated the presence of a heterozygous *SPTLC2* variant in the proband (II-3) and its absence in the unaffected parents (I-1 and I-2).

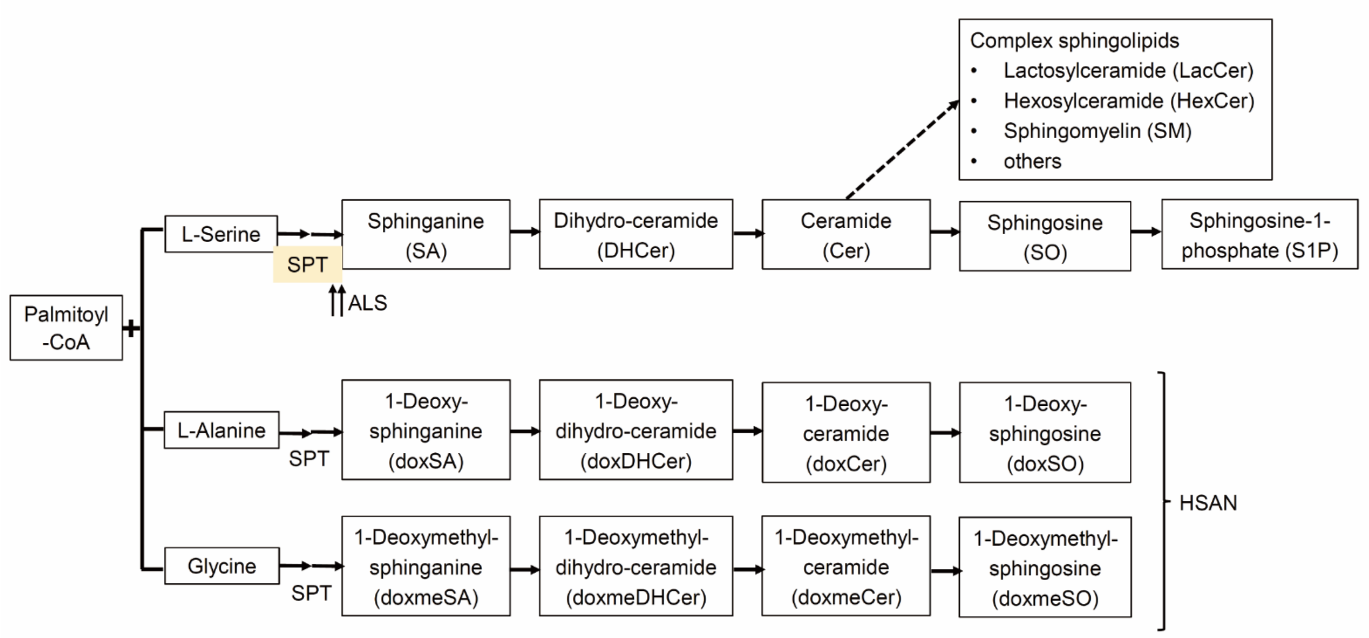

Supplementary Figure 4. Divergence of SPT enzyme pathways in HSAN1 and ALS

The illustrated SPT metabolic pathway shows different enzyme substrates, leading to diverse metabolic products. Variants that cause HSAN1 enhance SPT's preference for alanine and glycine, leading to the synthesis of deoxy-sphingolipids and deoxymethyl-sphingolipids. Conversely, ALS-causing variants increase the production of SA and Cer.

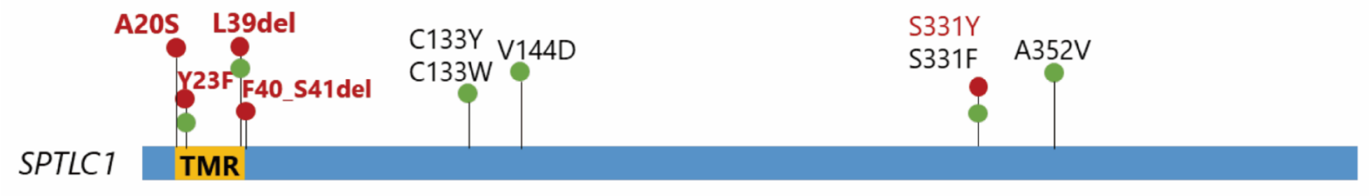

Supplementary Figure 5. Schematic representation of the SPTLC1 protein

Variants associated with early-onset ALS (red) are predominantly located in the transmembrane (TMR) domain of SPTLC1, in contrast to those associated with HSAN1 (green).

Supplementary Table 1. Candidate variants identified in pedigree 1 through the filtering steps

Gene	Chromosome	Location (GRCh37/hg19)	Nucleotide change [a]	Amino acid change	CADD score [b]	REVEL score [f]
NCAPG2 [c]	7	158486182	c.86C>A	p.Ala29Asp	18.2	0.065
RGS3 [d]	9	116346072	c.2380C>G	p.Pro794Ala	12.1	0.06
SPTLC2 [e]	14	78063644	c.212C>T	p.Ala71Val	23.0	0.458

All variants present in the affected individuals and absent in the unaffected individuals, in-house control participants, and public population databases based on the filtering step (Supplementary Fig 1) are shown.

a. All identified variants were heterozygous.

b. CADD scores for each variant were calculated based on the GRCh37-v1.6 model.

c. RefSeq ID: NM_017760

d. RefSeq ID: NM_017790

e. RefSeq ID: NM_004863

f. REVEL: Rare Exome Variant Ensemble Learner

Supplementary Table 2. *De novo* variants identified in pedigree 2 through the filtering steps of *de novo* analysis

Gene	Chromosome	Nucleotide change	Amino acid change	ToMMo 38KJPN AF	gnomAD Global AF
ADGRB2 [a]	1	c.1546G>A	p.Gly516Arg	ND	0.000013
KMT2C [b]	7	c.943G>A	p.Gly315Ser	0.0016	0.00043
SPTLC2 [c]	14	c.203T>G	p.Met68Arg	ND	ND

All candidate *de novo* variants with amino acid substitutions, each fulfilling the criterion of MAF <0.01 and identified based on the trio analysis (Methods), are shown. All the identified variants were heterozygous.

a. RefSeq ID: NM_001364857

b. RefSeq ID: NM_170606

c. RefSeq ID: NM_004863

AF, allele frequency; ND, not detected; gnomAD, The Genome Aggregation Database v3 (https://gnomad.broadinstitute.org/); ToMMo, Tohoku Medical Megabank Organization, jMorp (Japanese Multi Omics Reference Panel; https://jmorp.megabank.tohoku.ac.jp; 38KJPN)

Supplementary Table 3. Levels of sphingolipids in the plasma from the ALS-affected patients and controls in the pedigrees

Base forms (pmol/100μL plasma)									
Pedigree 1	d18:0 SA	d18:1 SO	d18:1 S1P	d20:1 S1P	doxSA	doxSO	doxmeSA	doxmeSO	
III-1 (unaffected)	0.12±0.05	1.32±0.03	68.08±1.74	0.96±0.04	ND	ND	0.13±0.003	ND	
III-2 (affected)	6.14±0.15	5.52±0.16	147.3±12.27	1.13±0.04	0.19±0.004	ND	0.15±0.016	ND	
IV-1 (affected)	2.19±0.08	4.00±0.20	115.7±10.55	0.89±0.02	0.07±0.005	ND	0.12±0.005	ND	
Pedigree 2									
I-1 (unaffected)	0.74±0.04	2.13±0.06	78.28±3.07	0.97±0.02	0.03±0.005	ND	ND	ND	
I-2 (unaffected)	1.85±0.05	3.43±0.14	91.49±5.90	0.92±0.10	ND	ND	ND	ND	
II-3 (affected)	3.30±0.04	3.59±0.07	113.7±3.92	1.08±0.05	0.06±0.001	ND	ND	ND	
Fatty-acid-acylated forms (nmol/100μL plasma)						(pmol/100μL plasma)			
Pedigree 1	Cer	DHCer	LacCer	HexCer	SM	doxDHCer	doxCer	doxmeDHCer	doxmeCer
III-1 (unaffected)	0.86±0.038	0.065±0.002	0.33±0.040	1.42±0.12	30.54±1.06	3.83±0.14	0.88±0.02	0.12±0.01	1.78±0.10
III-2 (affected)	2.79±0.086	0.26±0.015	0.39±0.089	1.90±0.16	44.22±5.64	17.06±0.78	3.10±0.06	0.51±0.03	4.24±0.26
IV-1 (affected)	2.72±0.30	0.23±0.014	0.43±0.040	2.63±0.26	39.64±3.13	9.57±1.25	1.61±0.04	0.56±0.16	3.94±0.66
Pedigree 2									
I-1 (unaffected)	0.78±0.081	0.032±0.002	0.35±0.007	1.44±0.088	30.52±6.45	5.71±0.17	1.32±0.07	0.15±0.03	1.65±0.18
I-2 (unaffected)	0.99±0.095	0.12±0.003	0.45±0.011	1.97±0.11	45.86±2.04	6.57±0.24	1.99±0.12	0.34±0.01	3.88±0.40
II-3 (affected)	1.76±0.084	0.13±0.008	0.52±0.030	2.16±0.055	29.43±0.42	7.14±0.36	2.60±0.13	0.39±0.03	2.37±0.16

Values represent the mean ± SEM (n = 3 or 4). The concentrations are shown as pmol/100μL or nmol/100μL on plasma samples. d20:0 SA and d20:1 SO were not detected in any of the samples. SA, sphinganine; SO, sphingosine; S1P, sphingosine-1-phosphate; doxSA, 1-deoxy-sphinganine; doxmeSA 1-deoxymethyl-sphinganine; doxSO, 1-deoxy-sphingosine; doxmeSO, 1-deoxymethyl-sphingosine; Cer, ceramide; DHCer, dihydroceramide; LacCer, lactosylceramide; HexCer, hexosylceramide; SM, sphingomyelin; doxDHCer, 1-deoxy-dihydroceramide; doxCer, 1-deoxy-ceramide; doxmeDHCer, 1-deoxymethyl-dihydroceramide; doxmeCer, 1-deoxymethyl-ceramide; ND, not detected.

Supplementary Table 4. Demographic and clinical features of patients with adult-onset ALS and controls screened for *SPTLC2* variants

	Patients with ALS*	Controls
	(n = 440)	(n = 1163)
Mean age at onset/sampling (SD)	63.5 (10.5)	49.2 (16.6)
Male-to-female ratio	1.40:1	0.87:1
Site of onset:		
Bulbar (%)	99 (28.0%)	NA
Spinal (%)	255 (72.0%)	NA
Familial disease (%)	40 (9.1%)	NA

The patients with ALS and the controls were of Japanese ancestry.

SD, standard deviation; NA, not applicable.

*Clinical data for age at onset (n = 24), sex (n = 24), and site of onset (n = 86) are missing.

Supplementary Table 5. *SPTLC2* variants identified in patients with adult-onset ALS and controls

Location (GRCh37/hg19)	Nucleotide change (NM_004863)	Amino acid change (NP_004854)	FALS (n=40)	SALS (n=400)	Control (n=1,163)	ToMMo 38KJPN AF	gnomAD Global AF
78082895	c.28T>C	p.Cys10Arg	0	0	1	ND	0.000007
78082883	c.40G>C	p.Val14Leu	0	0	1	0.00019	ND
78082873	c.50A>G	p.Asn17Ser	0	1	0	ND	ND
78045345	c.435G>T	p.Arg145Ser	0	0	1	ND	0.00006*
78043242	c.499A>G	p.Ile167Val	0	1	0	0.00012	0.000008*

All the identified variants were heterozygous.

FALS, familial ALS; SALS, sporadic ALS; AF, allele frequency; ND, not detected; gnomAD, The Genome Aggregation Database v3 (https://gnomad.broadinstitute.org/); ToMMo, Tohoku Medical Megabank Organization, jMorp (Japanese Multi Omics Reference Panel; https://jmorp.megabank.tohoku.ac.jp; 38KJPN).

*The variant was absent in gnomAD v3, but present in gnomAD v2; thus, the AF in gnomAD v2 for the variant is indicated.
